# Leadless pacing in peri-procedural settings

**DOI:** 10.1093/eurheartjsupp/suae093

**Published:** 2025-03-24

**Authors:** Mikhael F El-Chami, Ryan Cunnane

**Affiliations:** Division of Cardiology, Section of Electrophysiology, Emory University School of Medicine, 550 Peachtree Street, NE Atlanta, GA 30308, USA; Division of Cardiology, Section of Electrophysiology, University of Michigan, Ann Arbor, MI, USA

**Keywords:** Leadless pacemaker, TAVR, TTVR

## Abstract

Leadless pacemakers’ (LPs) main advantage over the traditional transvenous permanent pacemakers (TV-PPMs) is the absence of leads and subcutaneous pocket. These intracardiac pacemakers have been shown in observational studies to reduce long-term complications as compared with TV-PPM mainly by reducing the need for re-intervention. Two major advantages of this technology are the lower rate of infection (absence of pocket and lower rate of bacterial seeding) and lead-related complications (dislodgment). Hence, these advantages are more accentuated after transcutaneous valve replacement or valve surgery and clinical situations where it is important to reduce systemic infections and endocarditis. In this review, we highlight the role of LP in patients after transcutaneous and surgical valve replacements.

## Introduction

Leadless pacemakers (LPs) are an established alternative to traditional transvenous (TV) permanent pacemakers (TV-PPMs).^1–3^ The main advantage of these intracardiac devices is the lower rate of infection and the absence of TV leads, hence eliminating lead-related complications.^4,5^ Therefore, the use of these devices in clinical situations where their advantages are highly desired is warranted. The use of LP in patients with prior device infection at the time of, or shortly after, TV lead extraction (TLE)^6–8^ or in patients who require pacing after transcatheter valve replacement [transcatheter aortic valve replacement (TAVR) or transcatheter tricuspid valve replacement (TTVR)]^9,10^ and after cardiac surgery^10^ are some examples of these clinical scenarios that would greatly benefit from this technology. In this review, we will highlight the role of LP in a peri-procedural setting.

## Leadless pacemaker after valve replacement

### Leadless pacemaker after transcatheter aortic valve replacement and transcatheter tricuspid valve replacement

**Figure 1:**
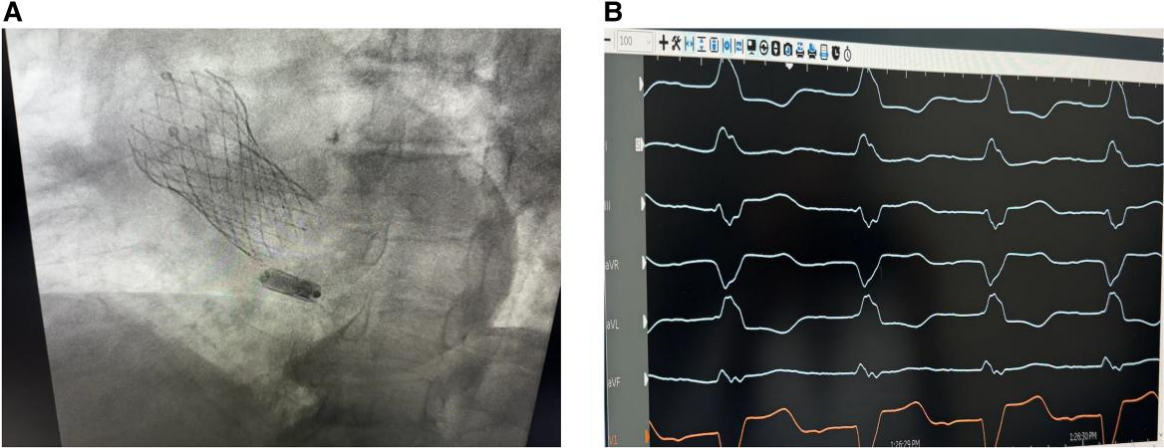
(*A*, *B*) Micra leadless pacemaker implanted in a post-transcatheter aortic valve replacement patient (*A*) with left bundle branch block and first-degree atrio-ventricular block (*B*).

Conduction system disturbances are common after transcatheter valve replacement and valve surgery. For instance, pacemaker implantation after TAVR or transcatheter aortic valve implantation is reported anywhere from 7 to 21%.^11–13^ Also, new-onset left bundle branch block after TAVR is commonly occurring in 11–16% of patients.^14^ A situation might warrant pacemaker implantation with no clear consensus of when to commit to pacing despite the higher risk of progression of conduction system disease (*Figure 1A and B*).^15^

Another transcatheter valve procedure that is gaining traction is TTVR. A procedure with high risk of conduction disturbances, for instance, pacing, was required in 25% of patients after TTVR with the Triscend II trial.^16^ In those patients, the presence of a large prosthetic tricuspid valve preferentially leads to a choice of tricuspid-sparing pacing system, i.e. a coronary sinus lead vs. a LP. A TV lead crossing this large prosthesis might lead to tricuspid stenosis, leaflet impingement, or valve thrombosis and malfunction over time.

The major advantage of a LP in this setting is the lower risk of infection, which would be devastating in patients with newly implanted transcatheter valve due to the risk of seeding.

**Figure 2:**
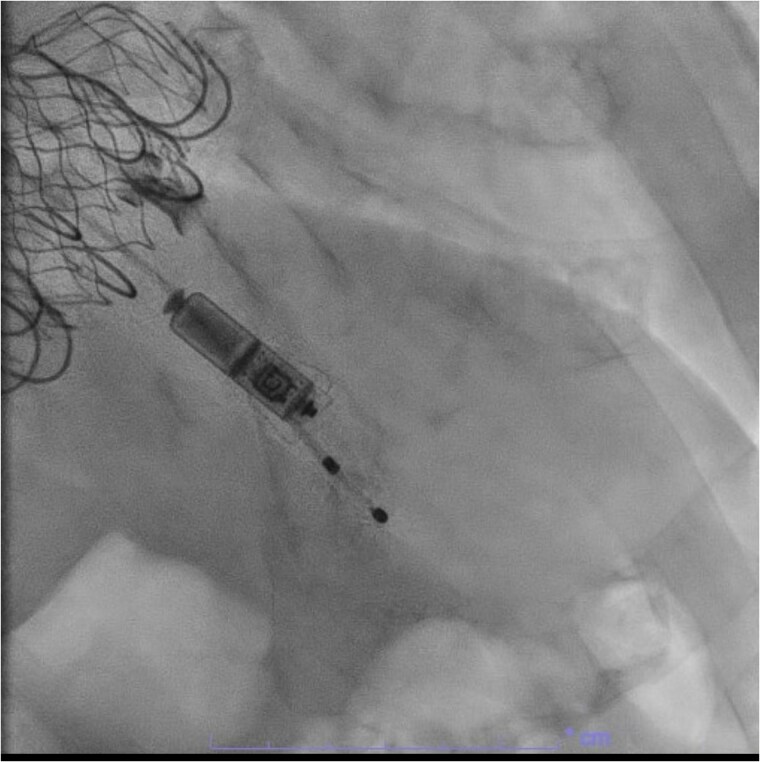
An apically placed Micra leadless pacemaker in a patient post-transcatheter tricuspid valve replacement and heart block.

**Figure 3:**
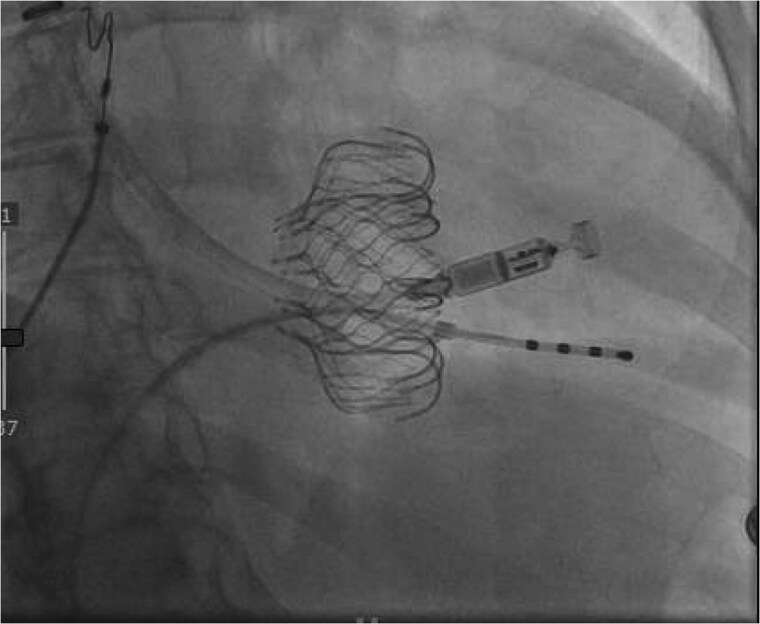
An Micra placed across the transcatheter tricuspid valve replacement via the internal jugular approach, also noted is a temporary pacemaker placed via the femoral approach.

From our experience, crossing the valve with a large delivery system is not simple. One must be careful and check in two orthogonal X-ray views, while extra caution is exercised to avoid dislodging and moving the valve. In addition, implanting a LP via the femoral route in the presence of a large transcatheter valve makes a septal implant challenging and often one must settle for an apical Implant (*Figure 2*). An advantage of an apical implant as compared with a mid-septal is that there is less chance to interact with the valve. Obviously, one has to accept the higher risk of perforation in that location. A jugular approach might be easier in these patients and would direct the delivery system preferentially to the septum (*Figure 3*). Furthermore, a shorter LP might be better in this setting to avoid interaction with the transcatheter TV that often extends deep into the RV.

Patients who undergo valve replacement surgery are also at increased risk of needing a pacemaker postoperatively. For instance, the risk of pacemaker implantation after AVR is 6.6%, after mitral valve replacement (MVR) is 10.5%, after combined AVR and MVR is around 13.3%.^17^ The risk after tricuspid valve replacement is even higher and reported at around 27%.^18^ Leadless pacemaker offers a good pacing option in these patients. The low risk of dislodgment and infection is a major advantage. Also, the ability to provide back-up pacing without having a permanent lead crossing the valve is important in patients with tricuspid valve replacement and also in patients with tricuspid valve regurgitation, commonly encountered in patients with mitral valve disease.

Oates *et al*.^19^ reported on their experience with intra-operative Micra LP implantation at the time of valve surgery in 100 consecutive patients undergoing valve surgery predominantly involving the tricuspid valve. These patients were deemed at high risk of post-operative conduction system disturbances due to the intended TV or multi-valve surgery and in some due to the presence of preoperative bradyarrhythmia. No complications were seen during follow-up.

This approach will add another layer of safety to the procedure since the LP is implanted under direct visualization, hence reducing/eliminating the risk of perforation and avoiding the use of a femoral venous route therefore eliminating venous access-related complications.

### Leadless pacing in cardiac implantable electronic device infection

The incidence of TV cardiac implantable electronic device (CIED) infection has been on the rise in recent decades as systems get more complex, indications expand, and implant populations get older.^20–22^ Leadless pacemakers, however, have demonstrated a low risk for systemic infection and device-related endocarditis.^23,24^ Combining data from the Micra LP pivotal study and post-approval registry documented no system-related infections out of 2520 implanted devices.^1,2^ Additionally, during the follow-up period in the pivotal study, a total of 16 patients developed documented bacteraemia or endocarditis.^25^ In this group, there were no documented device-related vegetations and all responded to antibiotics with no persistent bacteraemia after cessation of antibiotic treatment and no need for system removal. In real-world experience, the Micra LP has been implanted in tens of thousands of patients worldwide since Conformité Européene mark approval in 2015 and Food and Drug Administration approval in 2016. During that period, device-related infection has remained a rare reportable event with only a few case reports in the literature.^26–28^ While in prospective registries, no Micra infections have been reported, in the Micra CED 3-year follow-up, 6219 Micra devices were implanted.^20^ The infection rate was <0.17%. Medicare censors any adverse event when the events are rare. For example, if 1–10 patients have infections, the number will be reported as <11. Extrapolating from this data, Micra LP infections probably occur in 0.016–0.17% of implants.

A number of potential mechanisms have been proposed for why LPs present such a low risk for infection. One potential advantage is the miniature, self-contained design of the system itself. Without the need for a subcutaneous pocket or long TV leads, LPs have a much smaller surface area for bacteria to potentially seed.^23^ The Micra LP is also coated with parylene, which has demonstrated a reduced ability for bacterial growth on its surface relative to the bare titanium and polyurethane/silicone that make up the bulk of TV pulse generator surface and lead insulation, respectively.^29^ Device encapsulation and the fluid dynamics of the right ventricle have also been proposed as protective mechanisms against infection.^23^

As a result of the compelling data on LP resistance to infection, there has been a significant interest in their use in the setting of TLE for infected CIEDs. Infection is a clear indication for total system removal and consensus documents from the European Heart Rhythm Association^30^ and Heart Rhythm Society^31^ both recommend waiting at least 72 h after cultures have cleared before re-implantation after extraction of an infected TV system. This presents a particular challenge for patients who are pacemaker dependent, often leading to temporary externalized pacing and prolonged stays in the intensive care unit. Leadless pacemakers offer a more stable, and potentially permanent, solution to this common problem.

To date, Beccarino *et al*. presented the largest cohort and longest follow-up with 86 patients who underwent concomitant TLE for infection and LP implantation for ongoing pacing needs.^32^ Seventy-six percent had bacteraemia or fungaemia as their indication for TLE and of those 32% had vegetations and 38% had positive cultures within 72 h of the extraction/LP implant procedure. The LP was implanted before leads were removed in all patients, and the presence of leads did not impact the ability to successfully deploy the LP. There were no LP dislodgements related to the extraction procedure itself. Patients who present with CIED infection often have significant morbidities and that is reflected in the 29% mortality observed during follow-up, there was no procedure-related mortality. A total of 66.7% of those who did survive to discharge were able to complete their antibiotic treatment as outpatients. Multiple other small studies support the finding of a low risk of recurrent infection after LP implantation in the setting of TLE for infection.^33–35^

**Figure 4:**
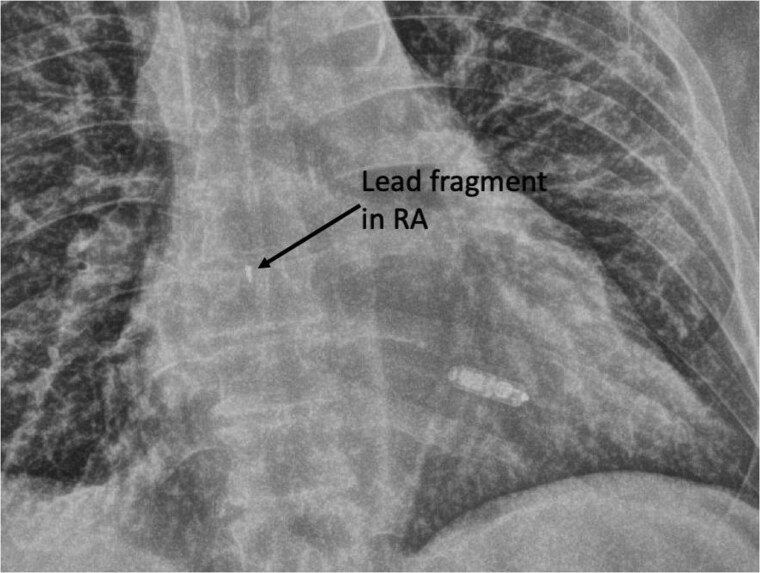
A Micra pacemaker implanted after transvenous lead extraction of an infected transvenous pacemaker. A retained atrial lead tip is noted in the right atrium. RA, right atrium.

A significant portion of patients who require LP support following TLE continue to have indications for more advanced therapies, like defibrillators or cardiac resynchronization therapy (CRT), that current LPs cannot provide. In this scenario, LPs can serve as a pacing bridge to allow for clearance of the infection prior to implantation of their destination device. In the Beccarino cohort, 14 patients were later upgraded to dual-chamber pacemakers, Micra AV systems, CRT defibrillators, or CRT pacemakers.^32^ Ten of these also had the LP removed at the time of upgrade with no related complications. In light of these findings, the advent of LPs allows the extracting physician the flexibility to offer durable pacing support that can facilitate the removal of the infected system while still meeting the pacing demands of the patient with minimal risk of persistent bacteraemia or recurrent infection. The LP can then serve as a destination therapy or a bridge to more advanced options while completing a full course of antibiotics (*Figure 4*).

While LP is a more costly therapy than the traditional TV pacemaker, the reported decrease in complications might make leadless pacing a cost-effective alternative.^21^

## Conclusion

Leadless pacemakers represent a significant innovation in the world of cardiac pacing and will continue to expand in their functionality and potential applications as the technology evolves. The unique features of these devices provide innovative solutions to many of the peri-procedural pacing problems facing implanting physicians today.

## Data Availability

The data underlying this article will be shared on reasonable request to the corresponding author.
